# Assessing the Positive Predictive Value of International Classification of Diseases Code K55.0 for Identifying Acute Mesenteric Ischaemia

**DOI:** 10.3390/diagnostics16203404

**Published:** 2026-10-20

**Authors:** Meis Jawad, Yasmin Soltanzadeh-Naderi, Talha Butt, Stefan Acosta

**Affiliations:** 1 Department of Clinical Sciences, Lund University, 202 13 Malmoe, Sweden; 2 Vascular Centre, Department of Cardiothoracic and Vascular Surgery, Skåne University Hospital, 214 28 Malmoe, Sweden

**Keywords:** acute mesenteric ischaemia, International Classification of Diseases, ICD-10, diagnosis, positive predictive value

## Abstract

**Background:** Acute mesenteric ischaemia (AMI) includes acute superior mesenteric artery (SMA) thrombosis and embolism, venous AMI, and non-occlusive mesenteric ischaemia (NOMI). Patients with AMI are identified using the International Classification of Diseases (tenth revision (ICD-10)) code K55.0 (acute vascular disorders of the intestine). Reporting of outcomes depends on reliable case identification. **Methods:** A retrospective review of all patients assigned with ICD-10 code K55.0 at a tertiary university hospital between 1st January 2023 and 31st December 2025 was performed. Medical records and imaging findings were scrutinized to validate the diagnosis. **Results:** Among 218 patients assigned ICD-10 code K55.0, 117 (53.7%) had confirmed AMI and 198 (90.8%) had acute gastrointestinal ischaemia. Patients with NOMI (*n* = 30, 53.3%) had a higher bowel resection rate than patients with embolic SMA occlusion (*n* = 21, 23.8%; *p* = 0.039) or venous AMI (*n* = 15, 6.7%; *p* = 0.012). The in-hospital mortality rate was lower in patients with venous AMI (6.7%) than in patients with NOMI (66.7%; *p* < 0.003) or thromboembolic SMA occlusion (42.9%; *p* = 0.015). **Conclusions:** The positive predictive value for AMI using ICD-10 code K55.0 was 53.7%, underscoring the need for validated case identification. The heterogeneity of diagnoses captured by this code supports consideration of a more granular ICD coding system. A conceptual proposal is presented for further discussion and validation.

## 1. Introduction

Acute mesenteric ischaemia (AMI) most commonly encompasses acute thromboembolic occlusion of the superior mesenteric artery (SMA), venous AMI, and non-occlusive mesenteric ischaemia (NOMI). The circulation to the intestines is abruptly impaired, leading to intestinal infarction, peritonitis, and death within a few days if left untreated. Historically, AMI has been difficult to diagnose, and most patients in the 1970s and 1980s were encountered at autopsy [1]. There has been no improvement in early diagnosis by clinical evaluation or plasma biomarkers [2]. However, the development of high-resolution computed tomography (CT) of the abdomen with intravenous contrast enhancement and imaging in the arterial and venous phase has greatly contributed to earlier diagnosis. CT angiography (CTA) can accurately describe the location of the mesenteric arterial and venous occlusion; can suggest if the occlusion/stenosis is due to embolism, thrombosis or dissection; and can evaluate signs of intestinal ischaemia, such as contrast defects of the bowel wall, intestinal dilatation, bowel wall thickening, or pneumatosis intestinalis, throughout the gastrointestinal tract [3]. Nevertheless, mortality remains high.

International Classification of Diseases (ICD) codes are widely used for tracking diseases within a patient population in in- or outpatient settings [4]. AMI has traditionally been coded with ICD-10 code K55.0. However, a recent validation study of a sample of patients diagnosed with this code between 2010 and 2022 showed that only 62.3% of patients assigned ICD-10 code K55.0 had AMI [5]. Assessing the positive predictive value of ICD-10 code K55.0 is therefore important when using this code to identify patients with AMI in clinical research and registry-based studies. The primary aim of the present study was to evaluate the positive predictive value of ICD-10 code K55.0 in a consecutive contemporary cohort of patients assigned this code at a tertiary university hospital by scrutinizing clinical records and CT images. A secondary aim was to compare outcomes between AMI subtypes and other forms of acute gastrointestinal ischemia to assess whether these clinically distinct entities warrant a more refined ICD classification.

## 2. Materials and Methods

### 2.1. Settings

Vascular surgery services for the southernmost part of Sweden are centralised to Skåne University hospital in Malmö, which serves as the tertiary referral hospital for approximately 2.14 million inhabitants (www.statistikdatabasen.scb.se; accessed on 3 August 2026). Patients with AMI were retrieved by conducting a search for patients admitted to Skåne University Hospital, located in both Malmoe and Lund, between 1 January 2023 and 31 December 2025, who were assigned the ICD-10 diagnosis codes K55.0 (acute vascular disorders of intestine) or I74.8 (embolism and thrombosis of other arteries) in either an inpatient or outpatient setting. Swedish law requires ICD-10 coding for all inpatient and specialized hospital care encounters, and funding is partly dependent on direct ICD performance metrics. ICD-10-SE is used for patient statistics, healthcare registers, research, and quality monitoring [6]. Diagnosis and procedure codes also contribute to the Nordic diagnosis-related group (NordDRG) system, which groups healthcare contacts with similar clinical characteristics and resource requirements. DRGs are used for healthcare planning and, resource allocation, and, in part, for healthcare reimbursement [7]. A previous study used both diagnostic codes, K55.0 and I74.8, for retrieval of patients with AMI from the same hospital [8]. Patient records with these ICD-10 codes were reviewed in the electronic medical record system Melior. Records of a total of 253 patients were retrieved.

### 2.2. Classification of Aetiologies of AMI

Classification of the aetiologies of AMI, as well as the nature of the occlusion (thrombosis or embolism) was meticulously determined by a senior vascular surgeon (S.A.) using predefined criteria outlined below. All available CT images were carefully reviewed before determining the precise cause of AMI. CTA or angiography was judged as having the best diagnostic capability, followed by autopsy, operative findings without assessment of the mesenteric arterial tree, and clinical notes. On rare occasions, when CT had not been performed or was performed without intravenous contrast enhancement, surgical findings, and/or autopsy reports were used to assess the underlying aetiology. Patients with clinical suspicion of AMI only, with or without having a CT with intravenous contrast enhancement, was excluded from this study. The term acute gastrointestinal ischaemia was defined as any acute ischaemic condition affecting the gastrointestinal tract, such as AMI, colonic ischaemia, mechanical bowel obstruction with intestinal infarction, or necrotizing enterocolitis. NOMI was defined as shock or cardiac failure together with patent SMA on CTA or at autopsy with concomitant severe small bowel ischaemia and/or severe colonic ischaemia. Isolated colonic ischaemia in critically ill patients in shock was labelled as NOMI. The presence of SMA stenosis may coexist in NOMI. If the SMA had not been examined in the presence of intestinal ischaemia, the aetiology was labelled indeterminate mesenteric arterial ischaemia. Venous AMI was defined as clinically manifest intestinal hypoperfusion caused by thrombotic occlusion of the mesenteric venous circulation, leading to intestinal insult within hours to days if left untreated. Colonic ischaemia was defined as self-limited, often left-sided, ischaemia of the colon, which did not fulfil the diagnostic criteria for NOMI or AMI.

### 2.3. Classification of Arterial Embolus and Thrombus

The nature of native arterial occlusion, embolic or thrombotic, was primarily determined by the localisation of occlusion and the extent of atherosclerotic wall lesions on CTA [9], or by catheter angiography. Embolic occlusion often appears as an oval-shaped clot surrounded by contrast in a non-calcified arterial segment, whereas thrombotic occlusion usually appears as a clot superimposed on a heavily calcified occlusive lesion [3]. The second most important source of information was the findings at autopsy. The presence of synchronous embolism, atrial fibrillation, previous embolism, and no or inadequate anticoagulation therapy in patients with atrial fibrillation at onset of AMI suggested embolism [3].

### 2.4. Statistics

Comparisons of proportions of categorical variables between groups were calculated with univariate binary logistic regression and were expressed as odds ratios (ORs) with 95% confidence intervals (CIs) and *p*-values. A statistically significant *p*-value was defined as *p* < 0.05. Calculations were performed in Statistical Package for the Social Sciences (SPSS version 28.0; Armonk, NY, USA). The positive predictive value for AMI with 95% CI was calculated using GraphPad QuickCalcs (https://www.graphpad.com/quickcalcs/confinterval1/) (Prism 11.1.0) with the modified Wald method (accessed on 26 August 2026).

## 3. Results

### 3.1. Distribution of ICD-10 Codes K55.0 and I74.8

**Figure 1:**
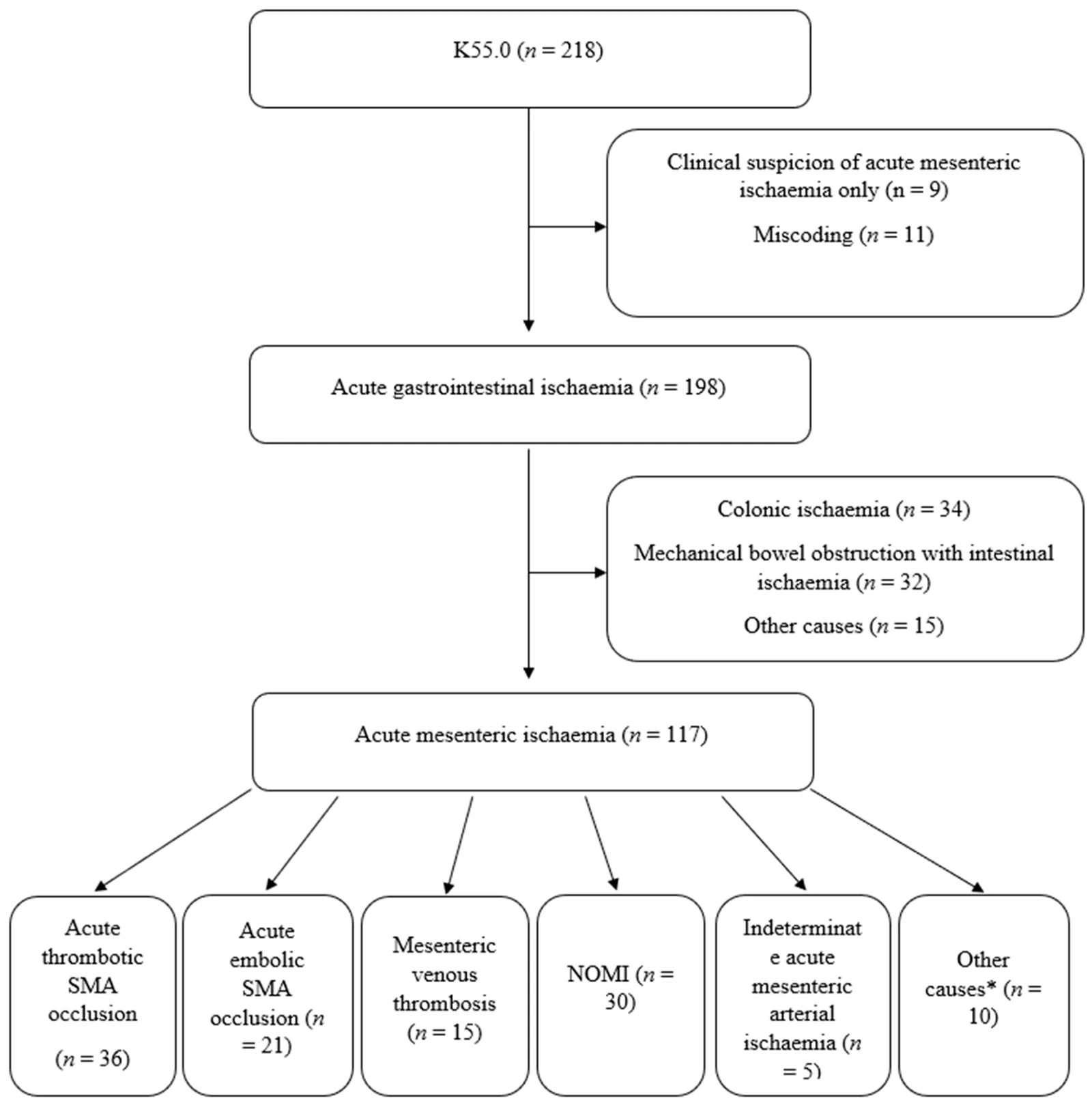
Breakdown of diagnoses of International Classification of Disease-10 code K55.0. SMA—superior mesenteric artery; NOMI—non-occlusive mesenteric ischaemia. * Other causes: occlusion of an SMA stent graft (*n* = 5), acute type B aortic and SMA dissection (*n* = 2), occluded iliaco-SMA bypass (*n* = 1), embolisation after stenting of the SMA (*n* = 1), and high-grade stenosis of SMA stent (*n* = 1).

Distribution of ICD-10 code K55.0 is shown in Figure 1. Among 218 patients assigned ICD-10 code K55.0, 198 (90.8%) had acute gastrointestinal ischaemia and 117 (53.7%) had confirmed AMI. The positive predictive value for AMI using ICD-10 code K55.0 was 53.7% (95% CI 47.0–60.2%). Among 35 patients assigned ICD-10 code I74.8, no patient had confirmed gastrointestinal ischaemia. The positive predictive value for AMI using ICD-10 code K55.0 and I74.8 was 46.2% (95% CI 40.2–50.2). One patient had asymptomatic venous AMI.

### 3.2. Aetiologies of AMI

The distribution of AMI subtypes is shown in Figure 1 and Table 1. The most common subtypes of AMI were acute thrombotic occlusion of the SMA (*n* = 36; 30.8%), followed by NOMI (*n* = 30; 25.6%), acute embolic occlusion of the SMA (*n* = 21; 17.9%) and venous AMI (*n* = 15; 12.8%).

### 3.3. Diagnosis of AMI

CT with intravenous contrast enhancement was performed to diagnose AMI in all patients with the exception of one patient with NOMI and four patients with an indeterminate cause of acute mesenteric arterial ischaemia. One patient with NOMI was diagnosed at autopsy. Five patients were labelled as having indeterminate cause of acute mesenteric arterial ischaemia; none of these patients underwent CT with intravenous contrast enhancement, and in-hospital mortality was 100% (Table 1).

### 3.4. Outcomes of AMI

Patients with NOMI (*n* = 30, 53.3%) had a higher bowel resection rate than patients with embolic AMI (*n* = 21, 23.8%; OR 3.7 [95% CI 1.1–12.6]) or venous AMI (*n* = 15, 6.7%; OR 16.0 (95% CI 1.9–137.6) (Table 2). Patients with NOMI (66.7%; OR 28.0 [3.2–244.3]) and arterial thromboembolic AMI (49.1%; OR 13.5 [95% CI 1.7–109.7]) had higher in-hospital mortality rates compared to patients with venous AMI (6.7%) (Table 2).

### 3.5. Diagnosis Confirmed, but Not AMI

Thirty-four patients had colonic ischemia (33 left-sided, and one in the cecum; of these, 17 were confirmed by colonoscopy, and six underwent surgery, of whom five underwent bowel resection). Thirty-two patients had mechanical bowel obstruction with intestinal infarction, and the causes were: adhesions (*n* = 14), hernias (*n* = 8; groin hernia [*n* = 3], abdominal wall hernia [*n* = 2], parastomal hernia [*n* = 2], umbilical hernia [*n* = 1]), cancer-related bowel obstruction (*n* = 5), volvulus (*n* = 3; sigmoideum volvulus [*n* = 2], caecal volvulus [*n* = 1]), internal hernia after gastric bypass surgery (*n* = 2). Adhesive small bowel obstructions were, in addition to ICD-10 code K55.0, also assigned ICD-10 code K56.5 (*n* = 5; intestinal adhesions with obstructions), K56.7 (*n* = 5; ileus, unspecified), K56.6 (*n* = 3; other and unspecified intestinal obstruction), whereas one patient was not assigned any ICD-10 code between K56.0 to K56.9. For patients with colonic ischaemia, the bowel resection and in-hospital mortality rates were 14.7% and 17.6%, respectively, and these rates increased to 81.3% and 34.4%, respectively, in patients with mechanical bowel obstruction with intestinal infarction. Two premature infants with necrotizing enterocolitis were incorrectly assigned ICD-10 code K55.0, underwent bowel resection, and survived. One child (<18 years) with abdominal cancer developed venous AMI, which was treated with anticoagulation therapy alone.

### 3.6. Diagnosis Not Confirmed

Nine patients had clinical suspicion of AMI only. CT with intravenous contrast enhancement was performed in 66.7% of these patients. The bowel resection rate was 0%, and the in-hospital mortality rate was 55.6%.

### 3.7. Proposal for a Revised ICD Classification Within the K55 Category

We propose a revised classification of ICD code K55.0 into separate diagnostic codes (K55.0–K55.9) and assigning colonic ischaemia a separate ICD code (K55.3) (Table 3).

## 4. Discussion

The present study showed that half of the patients assigned ICD-10 code K55.0 had confirmed AMI. Consequently, studies reporting outcomes of AMI based solely on this administrative code, without case validation, are likely to be affected by substantial misclassification bias [10,11,12]. Validation of ICD-based case identification therefore appears necessary when using administrative data to study AMI. This limitation is expected to be even greater in studies reporting outcomes of individual AMI subtypes, where reliance on ICD-10 code K55.0 alone is likely to result in further misclassification. Precise identification of patients with AMI and its subtypes therefore requires validation through medical records and CT images [8,12,13,14]. Similar observations have been reported for several other cardiovascular diseases, emphasising the importance of validating ICD-based case identification before reliable conclusions can be drawn from administrative datasets [15,16,17,18,19,20,21,22,23].

Importantly, the present study assessed the positive predictive value for ICD-10 code K55.0 but not its sensitivity. Patients with AMI assigned other diagnostic codes were therefore not identified, and the proportion of all AMI cases captured by K55.0 remains unknown. As diagnostic methods and disease classification evolve, corresponding revisions of diagnostic coding systems are necessary, and several medical specialities have similarly called for revision of disease-specific diagnostic codes [24,25].

Accurate ICD coding of AMI may improve the reliability of registry-based research and comparability between studies. However, accurate coding requires clearly defined disease entities. International consensus on the terminology and nomenclature of acute gastrointestinal ischemia is therefore needed, as definitions have been inconsistent across disciplines and clinical settings. An international multidisciplinary consensus study addressing this issue is currently ongoing, and includes a representative from the research group of the present study. This ongoing work has inspired the conceptual proposal for a more granular ICD coding system. Differences in pathophysiology, management, bowel resection rates, and in-hospital mortality provide a rationale for considering separate diagnostic codes. Likewise, colonic ischaemia should be distinguished from AMI because it was common and associated with substantially better outcomes [26], supporting a separate diagnostic code.

Validation of ICD-based case identification is therefore necessary. This can be achieved using different approaches. For less common diseases such as AMI, a complete review of medical records and CT images is feasible, as demonstrated in the present study. Adding ICD-10 code I74.8 to K55.0 in the search algorithm did not improve case identification but instead reduced the positive predictive value to 46.2%. When a complete chart review is not feasible, for example in large nationwide administrative datasets or when historical records are unavailable, validation of a representative sample can be used to assess the positive predictive value of the case-identification algorithm and evaluate uncertainties in the complete dataset [5]. Machine learning, a form of artificial intelligence, has emerged as a potential tool for identifying complex patterns and relationships in large datasets [16]. However, implementation of such methods in routine healthcare data remains challenging because of practical, regulatory, and data-protection considerations. Future development and validation of machine-learning algorithms, potentially incorporating unstructured data such as clinical notes, radiology, and pathology reports, may nevertheless improve ICD-based case identification in AMI.

Mechanical bowel obstruction complicated by intestinal infarction was coded with K55.0 in 32 patients in the present study, illustrating that K55.0 captures conditions other than primary AMI. The eight patients with hernia complicated by intestinal infarction would be more appropriately classified within the K40–K46 category, while other causes of mechanical bowel obstruction may be represented within the K56 category.

Among the 14 patients with adhesive bowel obstruction with intestinal infarction who underwent bowel resection and who were assigned K55.0, all but one were also assigned ICD-10 codes K56.5–K56.7. Adhesive bowel obstruction is a common surgical emergency, and may be complicated by bowel ischemia requiring emergency surgery [27,28]. It is likely that a large proportion of patients with adhesive bowel obstruction with intestinal infarction were not identified by K55.0 in the present study. In a large cohort of 492 patients with adhesive acute small bowel obstruction identified by ICD-10 code K56.5, 103 (20.9%) patients had bowel necrosis requiring bowel resection, while 49.2% underwent adhesiolysis without bowel resection and 29.9% were treated conservatively [29]. However, patients identified through K56.5–K56.7 were not systematically reviewed in the present study. The frequency of intestinal infarction among patients assigned these codes, as well as their diagnostic accuracy for this condition, therefore remains unknown. Further validation of the K56 codes would be required before conclusions regarding their refinement can be drawn.

A limitation is that information on who assigned the ICD codes was not available. Coding practices and the level of clinical experience of those assigning diagnostic codes may vary between clinical settings and thus potentially influence coding errors. The impact of these factors on the present findings could therefore not be assessed. Another limitation is that nine patients diagnosed with AMI on clinical grounds only could not be confirmed; these were therefore excluded from further analysis. Even if five of these patients underwent a contrast-enhanced CT, no diagnosis could be established reliably. This may be due to heterogeneity in contrast-enhanced CT protocols, including suboptimal imaging protocols, and/or difficulties in reliably detecting ischaemic bowel lesions in patients with NOMI. The distinction between embolic and thrombotic occlusion relies mainly on retrospective review of CTA findings. In patients with atherosclerotic mesenteric arteries, it may not always be possible to determine reliably whether an acute occlusion represents thrombosis superimposed on atherosclerotic disease or an embolic event. Heterogeneity of CTA protocols might have introduced difficulties in interpreting the nature of occlusion in some cases. Five patients were classified as having indeterminate acute mesenteric arterial ischaemia, as it was not possible in these cases to determine whether a thromboembolic occlusive event had occurred. Diagnostic validation by two independent reviewers and assessment of interobserver agreement was not performed, which may have introduced classification bias. Nevertheless, a strength of the study was the comprehensive validation of each patient retrieved from the hospital’s patient records, CT images and colonoscopy. Coding practices differ across countries [30]. For instance, Germany uses the German Modification of ICD-10 (ICD-10-GM)), which includes specific severity modifiers and rigid local documentation rules tied to financial throughput and DRG (Diagnosis-Related Group) system tariffs, driving clinical documentation behaviour. The present study did not assess whether the observed coding patterns were influenced by administrative or financial factors. The study results are therefore not easily transferable to other settings.

## 5. Conclusions

In conclusion, the positive predictive value of ICD-10 code K55.0 for identifying AMI was only 53.7%, and less than 20% for each individual AMI subtype, underscoring the importance of validated case identification. A more granular ICD classification distinguishing AMI subtypes and colonic ischaemia, including clearer guidance on coding for mechanical bowel obstruction, seems warranted. The proposed subcategories within the K55 category should be regarded as a conceptual proposal requiring multidisciplinary consensus and further validation.

## Figures and Tables

**Table 1:** Diagnosis, treatment, and outcomes in patients registered with ICD-10 code K55.0 (Acute mesenteric ischaemia).

	Number of Patients	CT with Intravenous Contrast	Endovascular Therapy	Bowel Resection	In-Hospital Mortality
**ICD code-10 K550**	218	204 (93.6)	40 (18.3)	87 (39.9)	85 (39.0)
**Acute gastrointestinal ischaemia**	198	189 (95.5)	40 (20.2)	85 (42.9)	79 (39.9)
**Diagnosis confirmed**					
**Acute mesenteric ischaemia (%)**	117 (53.7)	112 (95.7)	40 (34.2)	42 (35.9)	57 (48.7)
Acute thrombotic occlusion of the SMA (%)	36	36 (100.0)	18 (50.0)	13 (36.1)	19 (52.8)
Acute embolic occlusion of the SMA (%)	21	21 (100.0)	13 (61.9)	5 (23.8)	9 (42.9)
Venous AMI (%)	15	15 (100.0)	0 (0.0)	1 (6.7)	1 (6.7)
NOMI (%)	30	29 (96.7)	1 (3.3)	16 (53.3)	20 (66.7)
Indeterminate acute mesenteric arterial ischaemia (%)	5	0 (0.0)	0 (0.0)	2 (40.0)	5 (100.0)
Other causes (see Figure 1 for details of subtype) (%)	10	10 (100)	-	-	-
**Diagnosis confirmed, but not acute mesenteric ischaemia**	81				
Colonic ischaemia (%)	34	30 (88.8)	0 (0.0)	5 (14.7)	6 (17.6)
Mechanical bowel obstruction with intestinal ischaemia (%)	32	32 (100.0)	0 (0.0)	26 (81.3)	11 (34.4)
Other causes (%)	15				
**Diagnosis not confirmed or miscoded**					
Clinical suspicion only of acute mesenteric ischaemia (%)	9	6 (66.7)	0 (0.0)	0 (0.0)	5 (55.6)
Miscoding (not K550)	11				

ICD—International Classification of Diseases; SMA—superior mesenteric artery; NOMI—non-occlusive mesenteric ischaemia; CT—computed tomography.

**Table 2:** Differences in outcomes between different AMI subtypes.

		OR (95% CI); *p* Value			
	AMI Subtypes	NOMI vs. Thrombotic AMI	NOMI vs. Embolic AMI	NOMI vs. Venous AMI	Arterial Thromboembolic AMI vs. Venous AMI
Variable					
Outcomes					
Bowel resection		OR 2.0 (0.8–5.4) *p* = 0.16	OR 3.7 (1.1–12.6) *p* = 0.039	OR 16.0 (1.9–137.6) *p* = 0.012	OR 6.5 (0.8–53.0) *p* = 0.082
In-hospital mortality		OR 1.8 (0.7–4.9) *p* = 0.25	OR 2.7 (0.8–8.4) *p* = 0.095	OR 28.0 (3.2–244.3) *p* = 0.003	OR 13.5 (1.7–109.7) *p* = 0.015

AMI—acute mesenteric ischaemia; NOMI—non-occlusive mesenteric ischaemia.

**Table 3:** Conceptual proposal for a more granular classification within the ICD code of K55 category.

Current ICD 10 Codes	Proposed ICD Codes
K55.0 Acute vascular disorders of the intestine	K55.0 Acute arterial occlusion of the SMA
K55.1 Chronic vascular disorders of the intestine	K55.01 Acute thrombotic occlusion of the SMA
K55.2 Angiodysplasia of the colon	K55.02 Acute embolic occlusion of the SMA
K55.3 Angiodysplasia of the small bowel	K55.1 Venous acute mesenteric ischaemia
K55.8 Other vascular disorders of intestine	K55.2 NOMI
	K55.3 Colonic ischaemia
	K55.4 Chronic vascular disorders of the intestine
	K55.6 Angiodysplasia of the colon
	K55.7 Necrotizing enterocolitis
K55.9 Vascular disorder of intestine, unspecified	K55.9 Vascular disorder of intestine, unspecified

ICD—International Classification of Diseases; SMA—superior mesenteric artery; NOMI—non-occlusive mesenteric ischaemia.

## Data Availability

The raw data supporting the conclusions of this article will be made available by the authors on request.
